# Non-equilibrium lattice dynamics of one-dimensional In chains on Si(111) upon ultrafast optical excitation

**DOI:** 10.1063/1.5016619

**Published:** 2018-03-26

**Authors:** T. Frigge, B. Hafke, T. Witte, B. Krenzer, M. Horn-von Hoegen

**Affiliations:** Department of Physics, University of Duisburg-Essen, Lotharstr. 1, 47057 Duisburg, Germany

## Abstract

The photoinduced structural dynamics of the atomic wire system on the Si(111)-In surface has been studied by ultrafast electron diffraction in reflection geometry. Upon intense fs-laser excitation, this system can be driven in around 1 ps from the insulating (8×2) reconstructed low temperature phase to a metastable metallic (4×1) reconstructed high temperature phase. Subsequent to the structural transition, the surface heats up on a 6 times slower timescale as determined from a transient Debye-Waller analysis of the diffraction spots. From a comparison with the structural response of the high temperature (4×1) phase, we conclude that electron-phonon coupling is responsible for the slow energy transfer from the excited electron system to the lattice. The significant difference in timescales is evidence that the photoinduced structural transition is non-thermally driven.

## I. INTRODUCTION

Fundamental properties of photoinduced structural changes in solids are often studied through excitation by intense fs-laser pulses providing insight into the non-equilibrium dynamics of such transitions through ultrafast diffraction techniques.^1–14^ Hereby, the electron system is initially excited and the non-equilibrium population of the electronic states causes a transient change of the potential energy surface (PES). This scenario may give rise to a displacive excitation of the atom motion resulting in changes of the atomic geometry in the unit cell. Subsequent to the electronic excitation, however, thermalization of the electronic subsystem sets in and the disordered core motion is excited: the lattice system becomes hot. This situation leaves us with the question of whether a photoinduced phase transition is thermally or non-thermally driven. There are few rare cases where it was possible to disentangle the two processes of electronic and thermal excitation simultaneously. Hereby, extreme temporal resolution was employed to identify the two contributions through their different temporal evolution.^9,13,15^ The structural transition was driven through displacive excitation which caused a transient change of the potential energy surface on a sub-ps timescale. Heating of the lattice and thermal excitation of such a phase transition, however, usually proceeds much slower within a few ps.^4,16–18^

Employing the quasi 1D atomic wire system formed by self-assembly on the indium (In) reconstructed Si(111) surface,^19–24^ we demonstrate in a time-resolved electron diffraction study how the transient temperature rise, which occurs subsequent to the optical excitation, can be quantitatively characterized by means of the Debye-Waller effect. The knowledge of the maximum temperature rise Δ*T*_max_ and its temporal evolution Δ*T*(*t*) is crucial for the categorization of the driven phase transition.

## II. EXPERIMENTAL SETUP AND SAMPLE PREPARATION

The experiments were performed under ultra-high vacuum conditions at a pressure of $p=2\times 10^{-10}\;\text{mbar}$. We employed a time-resolved reflection high-energy electron diffraction (RHEED) technique implemented in a conventional pump-probe scheme to follow the ultrafast structural dynamics.^25^ The electron energy was *E* = 30 keV at an angle of incidence of ϑ=1.7°. In this diffraction geometry, the sample surface is initially excited with 100 fs laser pulses at 1.55 eV photon energy (5 kHz repetition rate) and an ultrashort electron pulse is used to probe the lattice dynamics at variable time delays Δ*t.*^25–28^ While the optical excitation occurs under normal incidence, the electrons travelling at *c*_0_/3 (*c*_0_, speed of light) are scattered under gracing incidence on the sample. This causes an unintended linear evolving time delay between pump and probe pulses along the surface. This so-called velocity mismatch was compensated by a tilted pulse front scheme for the laser pump pulse^29,30^ ultimately leading to a temporal instrumental response function of the entire experimental setup of 350 fs (full width at half maximum).^13^ For the benefit of an improved signal-to-noise ratio, the number of electrons in the probe pulse was increased at the expense of a slightly reduced temporal resolution of about 1 ps. The incident pump fluence Φ was adjusted between 0.7 mJ/cm^2^ and 6.5 mJ/cm^2^ by means of a continuously rotatable *λ*/2-waveplate in combination with the grating of the pulse front tilter.^30^ The pump beam diameter had a width of 8 mm which is much larger than the sample width of 2 mm and ensures a homogeneous excitation of the entire probed sample area.

Si(111) samples were cut from a phosphorus doped wafer (miscut <0.1°, specific electrical resistance 0.6–1 Ω cm). Clean surfaces with a well-ordered (7×7)-reconstruction were prepared by short flash anneal cycles at 1250 °C. Indium was evaporated from an e-beam evaporator onto the Si(111) surface at a substrate temperature of 500 °C. The surface quality was checked by low-energy electron diffraction and RHEED.

## III. ATOMIC WIRE SYSTEM Si(111)(8 × 2) ↔ (4 × 1)-In

**FIG. 1.**
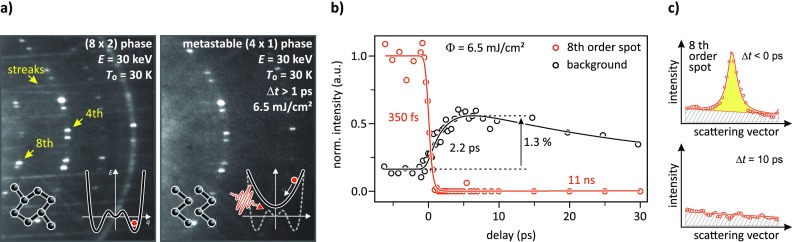
(a) Details of RHEED patterns of the indium reconstructed Si(111) surface prior (left) and shortly after optical excitation at delay times Δ*t* > 1 ps (right). The Si substrate temperature was *T*_0_ = 30 K and the electron energy *E* = 30 keV at an incidence angle of ϑ=1.7°. After photoexcitation, all signatures of the ground state vanish which indicates the structural (8×2)→(4×1) transformation. (This pattern has been rescaled for better visibility.) Insets depict the different atomic structures of both phases and the potential energy surface (PES). (b) Normalized intensity of an 8th order spot (red) and of the thermal diffuse background (grey) as a function of the delay time. While the 8th order spot disappears with a time constant of 350 fs, an increase in the background is observed on 6 times longer timescales. (c) Lineprofile through the 8th order spot prior (upper graph) and 10 ps after (lower graph) optical excitation. The solid line illustrates the data processing, where a Lorentzian fit with linear offset ensures a background (grey striped) subtracted determination of the spot intensity (yellow shaded). As becomes obvious, the spot is completely vanished after photoexcitation.

Adsorption of one monolayer (${\text{ML}}_{\text{Si}(111)}=6.24\times 10^{14}\;{\text{atoms}/\text{cm}}^{2}$) of indium results in the self-assembly of atomic wires on the Si(111) surface. This prototypical system exhibits a phase transition at *T_c_* = 130 K with doubling of the surface periodicity along and normal to the wires.^19–24^ In the low-temperature phase, the arrangement of the indium atoms is described by a distorted hexagon structure with (8 × 2) periodicity. Figure 1(a) (left panel) depicts the corresponding hexagonal structure and the RHEED pattern of this surface ground state at *T*_0_ = 30 K. The appearance of streaks rather than (×2) spots in the diffraction pattern is explained in terms of almost vanishing interchain coupling^31^ and is a typical signature of this ground state.^22^ The (8 × 2) reconstruction is due to the condensation of a charge density wave (CDW)^20,22,23^ where the distorted hexagonal arrangement of the surface atoms is directly linked to a bandgap of 0.1 eV in the electronic structure.^20,23,32^ Upon heating, a first-order insulator-to-metal transition is observed at about 125 K (Refs. 22 and 33–35) where the In atoms rearrange to metallic zig-zag chains with (4 × 1) periodicity.

Photoexcitation of the (8 × 2) low-temperature phase at fluences above 2 mJ/cm^2^ causes the complete disappearance of the diffraction spots at (8×) positions as well as (×2) streaks. This is apparent in the right panel of Fig. 1(a) which shows the corresponding RHEED pattern of the surface after optical excitation at delay times Δ*t* > 1 ps. For comparison, Fig. 1(c) shows lineprofiles through the marked 8th order spot prior (upper graph) and 10 ps after (lower graph) photoexcitation. As becomes obvious, the 8th order spots completely vanish upon photoexcitation: the surface undergoes an optically induced transition from the broken-symmetry (8×2) reconstructed ground state to a high symmetry state with (4 × 1) periodicity. The driving force for this transition is a non-thermal change of the potential energy surface (PES).^13^ At low temperatures and without optical excitation, the PES is essentially described by three distinct minima,^36^ with two equivalent minima of the ground state and one energetically excited minimum reflecting the (4×1) phase [compare inset of Fig. 1(a)]. Both structural phases are separated from each other by an energy barrier of about 40 meV.^37,38^ Shortly after the laser pulse excites the surface, the PES transiently changes^36^ and the surface undergoes a phase transition to the energetically favoured (4×1) structure on sub-ps timescales.^13^ Relevant for this transition are specific electronic excitations which couple to two vibrational eigenstates, commonly referred to as the soft shear mode and hexagon rotary mode. The linear combination of both describes the atomic motion upon the structural transformation. One fourth of their oscillatory period times of 1.2 ps and 1.8 ps serves as a good estimate for the transition time.^13,24,39^

To follow these dynamics, Fig. 1(b) shows the transient intensity of an 8th order spot (red) and the thermal diffuse background (grey). The first reflects the structural transition and the disappearance of the ground state signature is described by a time constant of *τ*_PT_ = 350 fs. In a previous study, the observed increase in the diffuse background at a time constant of *τ*_heat_ = 2.2 ps was explained in terms of a transient change of surface lattice temperature through multi-phonon losses of the diffracted electrons. Considering this and the significant different timescales finally led us to the conclusion that the phase transition is non-thermally driven.^13^ In the following, we will conclusively verify this statement and demonstrate how collective structural dynamics of driven phase transitions can be disentangled from incoherent lattice excitations by analyzing individual diffraction spots that are present in both phases. This further allows us to determine the temperature increase Δ*T*(*t*) of the metastable (4×1) phase for various fluences.

## IV. RESULTS

**FIG. 2.**
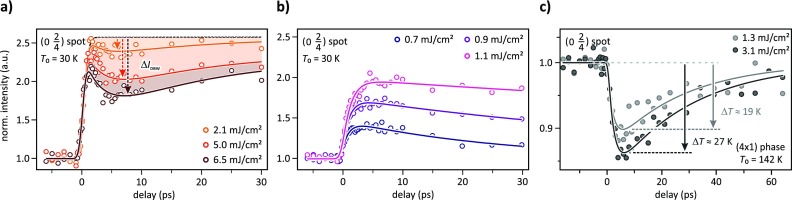
Transient intensity *I*(Δ*t*) as a function of the delay time for the $\left(0\frac{2}{4}\right)$ spot for different incident fluences ranging from 6.5 mJ/cm^2^ to 2.1 mJ/cm^2^ (a) and from 1.1 mJ/cm^2^ to 0.9 mJ/cm^2^ (b). At high fluences, the spots exhibit two competing processes: first, the intensity rises due to the structural transformation on a timescale of about 1 ps which is followed by a slower intensity loss and subsequent recovery on much longer timescales of 2.2 ps and 20–30 ps, respectively. (c) Transient intensity of the same spot at a substrate temperature of *T*_0_ = 142 K, i.e., above the critical temperature *T_c_*, for incident fluences of 1.3 mJ/cm^2^ and 3.1 mJ/cm^2^, respectively.

Upon the structural rearrangement of the surface atoms, most of the 4th order spots gain intensity due to structure factor enhancements in diffraction. This behavior is shown in Figs. 2(a) and 2(b) where the normalized intensity of the $\left(0\frac{2}{4}\right)$ spot was plotted as a function of the delay time Δ*t* for different incident laser fluences. Figure 2(a) depicts the dynamics I(Δt) for high incident fluences ranging from 2.1 mJ/cm^2^ to 6.5 mJ/cm^2^ and Fig. 2(b) for low incident fluences from 0.7 mJ/cm^2^ to 1.1 mJ/cm^2^. In the high excitation regime, the fast increase in I(Δt) is superimposed by a slower drop of intensity at Δ*t* ≈ 6 ps. The intensity of the $\left(0\frac{2}{4}\right)$ spot is thus subject to two competing processes.

The initial dynamics reflect the directed collective motion of the In atoms during the (8×2)→(4×1) structural transition. This accelerated displacive transition manifests in a very fast intensity increase by a factor of about 2.5 with a time constant of $\tau_{\text{PT}}\approx 350\;\text{fs}$ at high fluences. Thereafter, no further change of intensity is expected for this process [dashed line in Fig. 2(a)] since the metastable (4×1) phase survives for nanoseconds.^37,38^

In contrast, the second transient dynamics describe an exponential intensity loss with a 6 times longer time constant of *τ* = 2.2 ps that is followed by a recovery of intensity within 20–30 picoseconds. The transient minimum at 6 ps with a relative intensity loss of Δ*I*_DBW_ significantly scales with fluence, as can clearly be seen in Fig. 2(a). The observed timescales for excitation and relaxation are the same as those of the diffuse background^13^ providing evidence that this process reflects a transient temperature increase Δ*T*(*t*) of the metastable phase through a distinct temporary loss of intensity Δ*I*_DBW_ (*t*). Such behavior is explained by the Debye-Waller effect, which links the diffraction spot intensity *I*(*T*) to the momentum transfer **k** and the temperature dependent mean square displacement $〈u{\left(T\right)}^{2}〉$ of the surface atoms via

$$I(T)\propto \;\text{exp}\left(-\frac{{\left|k\right|}^{2}〈u{\left(T\right)}^{2}〉}{3}\right)\;.$$ (1)

**FIG. 3.**
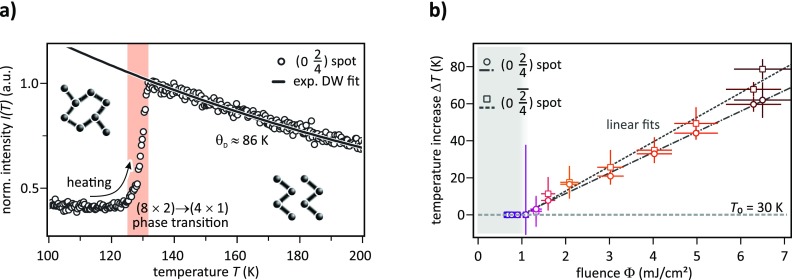
(a) Temperature dependent normalized intensity of the $\left(0\frac{2}{4}\right)$ spot during heating from 100 K to 200 K. The (8×2)→(4×1) phase transition is completed at 131 K. Fitting *I*(*T*) above 135 K yields a Debye-temperature of *θ_D_* ≈ 86 K for the (4×1) phase. (b) Transient temperature rise Δ*T* of two different 4th order spots as a function of the pump fluence for the metastable (4×1) phase at its maximum 6 ps after optical excitation.

Figure 3(a) depicts the temperature dependence of the $\left(0\frac{2}{4}\right)$ spot intensity *I*(*T*) upon increasing the substrate temperature from 100 K to 200 K in a (quasi-)static measurement, i.e., at a heating rate of *dT*/*dt* ≈ 0.07 K/s and without optical pumping. Upon the thermally induced structural (8×2)→(4×1) transition, the intensity also increases by a factor of about 2.5 within the temperature interval of 125 K and 131 K (shaded area). A further increase in temperature is accompanied by an exponential loss of intensity following Eq. (1). Accordingly, a fit of *I*(*T*) for *T* > 135 K (solid black line) yields a surface Debye-temperature

$$\theta_{D}=\sqrt{\frac{9\;\hbar^{2}\;T}{m\;k_{B}〈u{\left(T\right)}^{2}〉}}$$ (2)

of $\theta_{D}=\left(86\pm 11\right)K$, where *m* denotes the mass of the In atoms and *k_B_* the Boltzmann constant. This value for *θ_D_* is consistent with previously reported results.^40^

Comparing the thermally (see Fig. 3) and optically driven (see Fig. 2) phase transition scenarios reveals one important point: for the $\left(0\frac{2}{4}\right)$ spot as well as for all the other analyzed spots, a comparable structure factor enhancement was observed. This suggests that both phases, i.e., the thermodynamically stable (4×1) phase at *T*_0_ > 130 K and its metastable counterpart, are structurally identical. We thus employ the temperature dependent intensity *I*(*T*) as calibration to convert the transient intensity drop Δ*I*_DBW_ at 6 ps into a maximum temperature increase Δ*T*. These values are plotted in Fig. 3(b) as a function of the incident fluence Φ for the $\left(0\frac{2}{4}\right)$ spot (circles). Similar was done for the $\left(0\bar{\frac{2}{4}}\right)$ spot (squares) and within the uncertainty of measurement, both spots exhibit a consistent behavior. For the highest fluence of 6.5 mJ/cm^2^, the temperature rise at 6 ps is Δ*T* ≈ 80 K, i.e., from 30 K to 110 K maximum. Decreasing the excitation fluence leads to a linear decrease in Δ*T*_max_ as indicated in Fig. 3(b) by the two linear fits (dashed lines). Below a threshold value of about 1.1 mJ/cm^2^, no increase in surface temperature is observed.

## V. DISCUSSION AND CONCLUSIONS

In order to identify the origin of thermal lattice motion, we studied the excitation of the high temperature (4×1) phase at a substantial higher substrate temperature of *T*_0_ = 142 K which is well above *T_c_*. Figure 2(c) depicts the corresponding dynamic response of the $\left(0\frac{2}{4}\right)$ spot for two different incident fluences of 1.3 mJ/cm^2^ and 3.1 mJ/cm^2^. Upon photoexcitation, this spot loses intensity by 10% at 1.3 mJ/cm^2^, while a higher fluence results in a larger intensity drop. Apart from this, both low- and high-temperature measurements exhibit the same temporal evolution, i.e., the same excitation time constant of *τ*_heat_ = 2.2 ps and recovery time constant of *τ*_cool_ = 20–30 ps, independent of fluence.

At the temperature of 142 K, however, the surface has already undergone the (8×2)→(4×1) phase transition and the equilibrium surface structure is the metallic (4×1)-phase. Because no structural transition is involved, the observed intensity drops are only explainable by the Debye-Waller effect indicating an increase in incoherent lattice motion. Subsequent to the optical excitation, the electron system thermalizes and becomes hot, typically on a sub-ps timescale. Electron-phonon coupling then facilitates temperature equalization by transferring energy from the excited electron system to the lattice. The excitation of low-frequency modes which dominate the Debye-Waller effect is then observed at a time constant of 2.2 ps. Heat transport into the cold Si-substrate sets in on longer timescales. Converting the minimum intensity drop at 6 ps in Fig. 2(c) into a maximum temperature rise, we obtain values comparable to those plotted in Fig. 3(b). Since the transient intensity drop of the (4×1) spot at low temperatures of *T*_0_ = 30 K [Fig. 2(a)] and the dynamic response of the same spot in the high-temperature phase at *T*_0_ = 142 K [Fig. 2(c)] exhibit the same temporal evolution, we can finally conclude that the excitation of low-frequency modes is governed by electron-phonon coupling, while phonon-phonon scattering plays a minor role. This finding is in contrast to previous observations for a Peierls-distorted system.^41^

What remains striking is the fact that the temperature of the system does not increase for fluences below 1 mJ/cm^2^. The absorbed photon energy has to go somewhere and one possible explanation is the application of latent heat before the phase transformation sets in, which is a typical signature of a first-order phase transition.
